# The Effect of a Curved Non-Motorized Treadmill on Running Gait Length, Imbalance and Stride Angle

**DOI:** 10.3390/sports6030058

**Published:** 2018-06-29

**Authors:** Andrew Hatchett, Kaitlyn Armstrong, Brian Parr, Mallory Crews, Charlie Tant

**Affiliations:** Department of Exercise and Sport Sciences, University of South Carolina Aiken, Aiken, SC 29801, USA; kaitlyna@usca.edu (K.A.); brianp@usca.edu (B.P.); mecrews@usca.edu (M.C.); ctant@usca.edu (C.T.)

**Keywords:** treadmill, curved, running gait, imbalance, stride angle, stride length, step length

## Abstract

Running on a non-motorized, curved-deck treadmill is thought to improve gait mechanics. It is not known, though, whether the change in gait carries over to running on a motorized treadmill on level ground. To determine the effect of running on a curved non-motorized treadmill (CNT) on gait characteristics, measured during a subsequent bout of running on a traditional motorized treadmill (TMT), sixteen healthy college-aged participants, aged (mean ± SD) 20.4 ± 1.6 years, volunteered to have their gait analyzed while running on a TMT and CNT. After familiarization with, and a warm-up on, both treadmills, each subject completed five 4-min bouts of running, alternating between traditional motorized and curved non-motorized treadmills: TMT-1, CNT-1, TMT-2, CNT-2, and TMT-3. Variables of interest included step length (m), stride length (m), imbalance score (%), and stride angle (°), and were measured using Optogait gait analysis equipment. We found differences in gait characteristics among TMT-1, TMT-2, and TMT-3, which can be attributed to running on the CNT. The results show that running on a CNT resulted in significant changes in gait characteristics (step length, stride length, imbalance score and stride angle). These findings suggest that running on a CNT can significantly influence running gait.

## 1. Introduction 

Running has remained a popular exercise for decades all over the world. In the United States of America alone, it is estimated that over 16 million people finish running races annually [1]. According to some experts, long-distance running was crucial in creating our current upright body form [2]. Humans are one of the few species who have mastered bipedal locomotion, and their foot has evolved to be the basis for such a specialized gait [3]. The human foot alone comprises 26 bones, 33 joints and 19 muscles [3,4]. The bones are arranged to form a medial longitudinal arch, which makes it ideal for its function of supporting the weight of the body and spreading the forces experienced during gait [3,5]. As mentioned by Altman and Davis [6], analysis of rear foot striking (RFS) in a barefoot condition landing with RFS results in a very defined impact peak in the vertical ground reaction force during contact, which precedes the propulsion peak. This results in high loading rates in early stance. However, forefoot strikers are able to eliminate this impact through eccentric loading of the posterior calf musculature, which significantly reduces this loading. Midfoot striking results in more variable loading, but load rates typically fall between the rear foot and forefoot strike pattern. It has been suggested that the anatomy and small surface area of the heel is suited for the loads in walking, but not for attenuating the repeated impacts associated with running [6,7]. Even with the running shoe evolution, approximately 75% of shod runners experience heel strike [6]. Interestingly, a similar percentage of runners report injuries associated with running (up to 79% [7]). Forefoot striking while running takes greater advantage of the energy-storing capacity of the arches, which is evidenced by the increased vertical arch motion during load acceptance [6,8]. 

Humans began using treadmills as a mode of aerobic exercise in the 1960s. Since that time, treadmills have grown in popularity and sophistication. Treadmills allow users to walk, jog, and even run at a variety of speeds. As technology improved, designers began creating treadmills with the capacity to simulate walking or running up or down a hill by manipulating the incline. These improvements in the technology have led to the modern treadmill, which allows users to pick a predesigned workout program. These designed programs increase/decrease speed and incline at specified times throughout the exercise routine. Due to treadmill versatility, they have become one of the most widely used pieces of aerobic exercise equipment [9]. Motorized and non-motorized treadmills allow participants the convenience of training aerobically on a machine while staying in one place. Non-motorized treadmills have no motor and rely on the user’s energy to move the belt [9,10]. Due to total manual operation, participants can instantly adjust their pace with a few explosive steps. One evolution of the non-motorized treadmill has been a curved platform forming a curved non-motorized treadmill (CNT). Manufactures believe that the arced design inspires the user to run with a more mechanically efficient gait. If this is an accurate belief, certain aspects of a runner’s gait, such as foot strike pattern, stride length, stride angle and imbalance, may be retrained without advanced and costly laboratory equipment. 

There has been much preoccupation with foot strike pattern and associated biomechanical variables on running economy [11,12,13]. Mechanical factors can be divided into forces (kinetics) and movement patterns (kinematics). A runner exhibiting abnormalities in either of these areas can experience excessive loading on their musculoskeletal system [1]. Runners experiencing both excessive forces and abnormal movement patterns (gait) are likely to have an even greater risk of injury [14]. There are numerous components to gait, some of which include step length, stride length, imbalance or asymmetry and stride angle. A more compact step length and stride length are associated with a more efficient transfer of energy during running. A more symmetric running pattern indicates a more balanced application of force and therefore a more efficient effort. Stride angle correlates directly with running economy. Each of these variables is modifiable through feedback. The idea of altering gait patterns using feedback is not novel. The earliest forms of feedback were limb load monitors placed within the shoe of a patient [15,16,17,18]. The aim of this type of feedback was to produce an equal load distribution between the lower extremities during gait. Traditional gait retraining efforts occur on a motorized treadmill. When considering previous gait retraining research, and the belief that a curved non-motorized treadmill can stimulate a shift in running gait, it is logical that, if an athlete runs on a curved non-motorized treadmill, their gait pattern will change. Therefore, the purpose of this study was to determine if running on a curved non-motorized treadmill influences running gait. 

## 2. Methods 

### 2.1. Participants

Based upon an a priori power analysis, with α = 0.05 and β = 0.20, 16 volunteer participants were used in this research. Male and female recreationally trained athletes, between the ages of 18 and 60 years old, were solicited through advertisements. The participants had to be free of injury and capable of running for the required time period, as designated in the study guidelines (20 min). Prior to inclusion, all subjects provided informed consent according to the institutional review board of the University of South Carolina (Pro00060177). 

### 2.2. Procedures

The anthropomorphic measures (height, weight, age) of the participants were recorded before they engaged in any running effort. Once these preliminary data were collected, participants were given time to familiarize themselves with the two different types of treadmills. All participants had experience running on a traditional motorized treadmill (GE 2000 Series, General Electric Healthcare, Chicago, IL, USA). None of the participants had experience running on an arced non-motorized treadmill (Enduro, TrueForm Runner, Chester, CT, USA). Once the participants felt familiar with both treadmills (after approximately 5 min), a series of 4-min running bouts were completed. The first bout (TMT-1) was on a traditional treadmill. Then, the participant dismounted the traditional treadmill and immediately began a 4-min bout on the CNT. Following this bout of running, the participant moved back to the motorized treadmill (TMT-2), then back to the CNT, ending with a final 4-min bout on the traditional motorized treadmill (TMT-3): TMT-1, CNT-1, TMT-2, CNT-2, and TMT-3. The speed at which the participants ran during each bout was self-selected based upon the speed that they believed they could maintain on the CNT and the traditional motorized treadmill. Due to the non-flexible nature of the instrumentation, all gait data were collected on the traditional motorized treadmill.

### 2.3. Gait Variables

The variables of interest for this research included step length, stride length, imbalance and stride angle.

**Figure 1:**
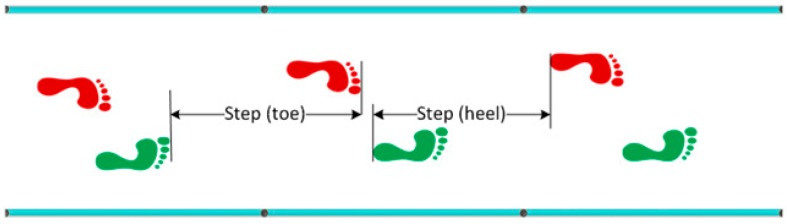
Step Length.

**Figure 2:**
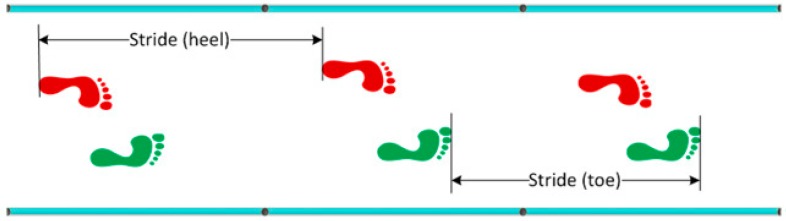
Stride Length.

**Figure 3:**
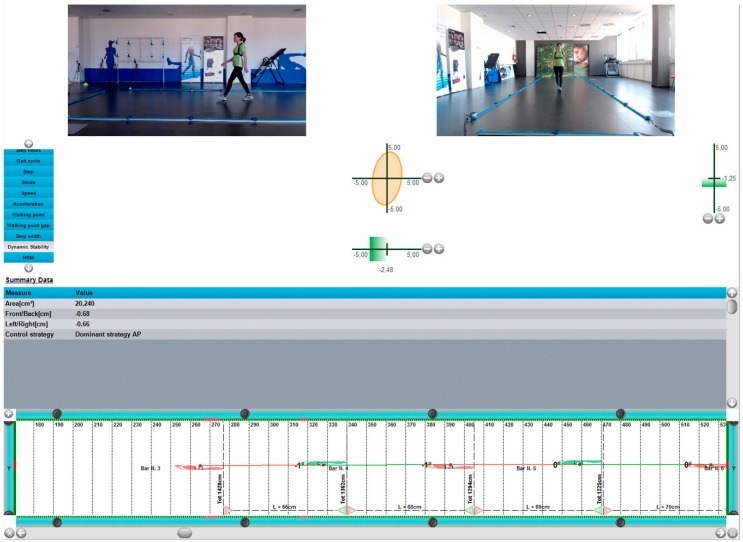
Imbalance.

**Figure 4:**
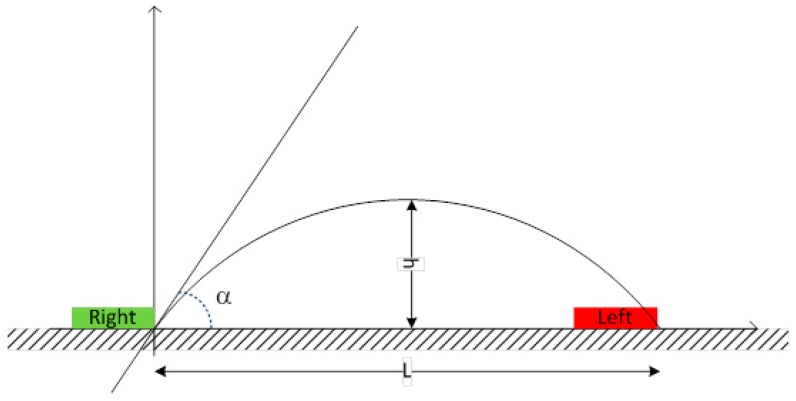
Stride Angle.

- Step length is the distance between the tip (toe) of two subsequent feet or the distance between the heel of two subsequent feet (measured in meters) (Figure 1).
- Stride length is defined as the distance between the tip of two subsequent footprints of the same foot or the distance between the heel of two subsequent footprints of the same foot (measured in meters) (Figure 2).
- Imbalance is an indicator of running ‘asymmetry’ between the right and the left legs. The difference between the ideal and real time, and the relation between the difference and the ideal time (expressed as a %), can be defined as imbalance (measured in degrees) (Figure 3).
- Stride angle is defined as the angle of the parable tangent derived from the movement of a stride (L = stride length, h = height to which the foot is risen) (measured in degrees) (Figure 4).

### 2.4. Instrumentation

The Optogait photoelectric cell system (Microgate, Bolzano, Italy) was used to measure gait variables (step length, stride length and stride angle). The Optogait system consists of two rigid parallel bars (a transmitter unit and a receiver). The bars were placed on either side of the traditional motorized treadmill, approximately 70 cm apart and parallel to each other. The rigid nature of the bars did not afford the opportunity to place them on the arced non-motorized treadmill. The Optogait system was connected (via USB) to a personal computer (Lenovo, model T 530, Lenovo, Morrisville, NC, USA). Optogait software (software version V1.10.7.0, Microgate, Bolzano, Italy) was used for quantification of all gait measurements. This system has been determined to be a valid instrument in the measurement of gait parameters [19].

Imbalance was measured using a Gyko inertial measurement tool. The Gyko inertial sensor system (Microgate, Bolzano, Italy) contains a three-dimensional accelerometer, gyroscope, and magnetometer which allows for recordings (full scale range: 8 g) at a sampling frequency of 500 Hz. The Gyko system was perpendicularly attached to an elastic belt, provided with the system. The Gyko system was fixed at the mid-scapular level on the back of the body (between the shoulder blades), as indicated by the manufacturer (http://www.gyko.it/en). During assessment, accelerometer and gyroscope signals were transferred via bluetooth to a personal computer and stored using the proprietary software (GykoRePower Software, Microgate, Bolzano, Italy). The Gyko accelerometer has been determined to be a valid instrument for the measurement of imbalance [20].

Treadmills used in this research effort included a traditional motorized treadmill and an arced non-motorized treadmill. The traditional motorized treadmill used in this research was a GE 2000 Series clinical grade treadmill set at a grade of 0 degrees. The arced non-motorized treadmill used in this research was a TrueForm Runner Enduro model (True Form Runner, Chester, CT, USA).

### 2.5. Statistical Analysis

Statistical analyses were conducted using the Statistical Package for the Social Sciences (SPSS) (Version 24, IBM Corp, Armonk, NY, USA). Normal distribution of the data was confirmed through a Shapiro–Wilks test and visual inspection of box plots, normal Q-Q plots, and frequency histograms. Descriptive statistics, including mean value and standard deviation (mean ± SD), were calculated for the physical characteristics and performance variables (Step Length, Stride Length, Imbalance and Stride Angle). Paired sample t-tests were conducted to determine if there were statistically significant differences between bouts of running as compared with baseline measures (TMT-1/TMT-2 and TMT-1/TMT-3). All calculations were performed with an a priori level of significance of *p* ≤ 0.05.

## 3. Results

### 3.1. Physical Characteristics

The physical characteristics of the study participants is offered in Table 1. The average age of the participants was 20.46 y (±1.69), height 172.33 cm (±7.17) and body mass 69.08 kg (±11.14). Included in Table 1 is a profile of Female and Male participants respectively.

### 3.2. Gait Performance Variables

Mean values for the gait performance variables for each respective 4-min trial are presented in Table 2. Variables were measured on a traditional motorized treadmill (TMT-1) prior to the participant experiencing the curved non-motorized treadmill, then immediately after a 4-min exposure to the curved non-motorized treadmill (TMT-2) and immediately following a second 4-min exposure to the curved non-motorized treadmill (TMT-3).

### 3.3. Statistical Analysis

Results from paired sample t-tests (Table 3) determined a statistically significant difference between all four of the variables of interest from TMT-1 to TMT-2. Additionally, a statistically significant difference between each of the four variables of interest from TMT-1 to TMT-3.

## 4. Discussion

Forward locomotion is a common, yet complex, movement pattern that has been analyzed since the time of Aristotle [21]. This locomotion follows a cycle of standing still, walking, running, and sprinting. The demarcation between walking and running occurs when periods of double support, during the stance phase of the gait cycle (both feet are simultaneously in contact with the ground), give way to two periods of double float at the beginning and the end of the swing phase of gait (neither foot is touching the ground). Generally, as speed increases, initial contact changes from the hind foot to the forefoot. Paradoxically, approximately 80% of distance runners are rear foot or heal strikers. Most of the remainder are considered midfoot strikers [22]. Interestingly, research has indicated that human gait patterns can be retrained (significantly influenced) from a less efficient or less safe pattern to a more efficient and safer pattern [15]. Previous research utilized a series of 8 sessions of retraining using real-time visual feedback. The aim of this study was to determine if running on an arced non-motorized treadmill significantly influenced running gait. The physical characteristic profile of the participants is presented in Table 1. While Table 2 reveals that short (4-min) bouts of running on an arced non-motorized treadmill can influence stride length, step length, imbalance and stride angle. Statistically significant differences in all four of the variables of interest are shown in Table 3 when comparing the results of trial 1 and trial 2, as well as those of trial 1 and trial 3.

Step length and stride length are invariably linked, in that a running stride length is equivalent to the step length with the addition of flight time. Therefore, the results offered in this study for the step length and stride length variables display an appropriate relationship. As the stride length decreases with greater exposure to the arced non-motorized treadmill, so does the step length. Research has indicated that a link exists between the stride length and impact characteristics, such that as the finding that stride length greatly increases impact [23,24,25]. A reduction in stride length, although it would appear smaller in trained runners, may be advantageous, as it has been shown to reduce impact peaks [26,27,28] and loading rates [28,29,30] experienced by runners. A shorter stride length means that the heel is located more underneath the center of mass (COM), which reduces the amount of hip and knee flexion required [31]. A more efficient running gait pattern leads to a reduction in stride length of 6–8% in inexperienced runners and those with a long history of running [26,27,28,29,30]. Schubert [31] indicated that an increased stride rate (decreased stride length) affects impact peak, kinematics, and kinetics, and therefore may be considered a mechanism with which to influence the injury risk and recovery of a runner. Specifically, similarities are seen across all studies, with a decreased center of mass vertical excursion, ground reaction force, impact shock and attenuation, and energy absorbed at the hip, knee, and ankle, as step rate is increased, or step length is decreased, at a constant speed [31].

Analysis of imbalance or asymmetry resulting from running on a CNT treadmill yielded statistical significance as well. A lack of symmetry, that is, relative differences in muscle strength, motion, flexibility, balance, and mechanics between sides of the body, is one element often highlighted as a risk factor for injury. The imbalance measure used in this research is an indicator of running ‘asymmetry’ between the right and left foot. A more symmetric running gait would indicate a more balanced athlete, and thus a more efficient athlete [32]. Nasirzade and colleagues [33] report that gait symmetry and limb coordination are necessary to achieve balanced movement. However, gait asymmetry is a condition brought about by internal or external abnormalities. When traveling at normal walking speed, most people have an asymmetric gait. A reason for this is that body segments act as pair oscillators, in which the symmetric relationships (in-phase and out-phase) are more easily maintained at higher speeds than under other complex phase conditions. For example, feet are more likely to experience non-coupling and apply different functional strategies at lower speeds, while motion patterns are carried out with higher coupling and symmetry at higher speeds [33,34]. The longer the time a respective foot spends in support of the body, the greater the asymmetry of the gait. This information is consistent with the results presented in this research. As step and stride length decreased, and speed stayed constant, the amount of time that either lower limb spent in support decreased accordingly, resulting in a decrease in imbalance. When examining the data, one can readily see a great decrease in the imbalance score from TMT-1 to TMT-2, as compared with that from TMT-1 to TMT-3. There was an increase in the imbalance score from TMT-2 to TMT-3. A possible explanation for this may be the fatigue of the participants at this stage of the study. However, further research is required to determine the actual cause for this change.

Stride angle is defined as the angle between the theoretical tangent (created from an arc drawn from the toe-off to the initial ground contact of the same foot) and the ground [35]. Stride angle is a biomechanical feature of gait analysis that correlates significantly with running economy [36]. Stride angle may be a marker of the athlete’s ability to efficiently maximize swing time and minimize contact time with effective energy transfer during ground contact [26]. Research has indicated that a mid/forefoot strike pattern with a stride angle of less than 4 degrees correlates with a better running economy [36]. Results from this research show that 4-min bouts of running on an arced non-motorized treadmill influence stride angle in a statistically significant manner. The mean trend for stride angle as a result of running on an arced non-motorized treadmill is indicative of better running economy via a decrease in contact time.

Limitations associated with the current research include a lack of standardizing the participants’ footwear. In an effort to have the participants maintain their most normal running form familiar footwear was used in this study. However, this footwear may also impact the participant’s gait pattern. Also, not controlling the running experience on treadmills may also be a limitation associated with this research effort. Additionally, not controlling for the participant’s level of fitness may be another limitation. Conceivably, the level of fitness of the participants could influence their ability to run for the twenty-minute period prescribed in this study’s protocol. 

## 5. Conclusions

Running on a curved non-motorized treadmill can have a significant influence on certain gait performance measures. Future research may consider examining the translation of curved non-motorized gait retraining in relation to real-world gait patterns. Additional research may include an examination of the duration of the effect and potential muscle recruitment pattern differences from the use of arced non-motorized treadmills.

## Figures and Tables

**Table 1:** Physical characteristics of female and male participants (mean ± SD).

Characteristic	Pooled (N = 16)	Female (n = 10)	Male (n = 6)
Age (y)	20.46 ± 1.69	20.06 ± 1.78	21.01 ± 1.67
Height (cm)	172.33 ± 7.17	167.28 ± 5.17	178.22 ± 3.74
Body Mass (kg)	69.08 ± 11.14	64.93 ± 9.38	75.99 ± 11.05

N: total study population; n: respective group population.

**Table 2:** Values of gait performance variables (mean ± SD).

Variable	TMT-1	TMT-2	TMT-3
Step length (m)	0.86 ± 0.08	0.75 ± 0.11	0.68 ± 0.08
Stride length (m)	1.89 ± 0.55	1.65 ± 0.19	1.52 ± 0.14
Imbalance (°)	−1.37 ± 2.65	−0.36 ± 2.13	−1.13 ± 1.75
Stride angle (°)	3.55 ± 4.39	1.23 ± 0.83	0.47 ± 0.17

**Table 3:** Results of Paired Sample t-tests to determine differences between performance variables from baseline.

Variable	TMT-1 vs. TMT-2	TMT-1 vs. TMT-3
Step length (m)	*p* = 0.039 *	*p* = 0.026 *
Stride length (m)	*p* = 0.033 *	*p* = 0.042 *
Imbalance (°)	*p* = 0.001 *	*p* = 0.007 *
Stride angle (°)	*p* = 0.001 *	*p* = 0.001 *

* Indicates statistically significant difference.
